# Microbiome composition within a sympatric species complex of intertidal isopods (*Jaera albifrons*)

**DOI:** 10.1371/journal.pone.0202212

**Published:** 2018-08-29

**Authors:** Marius A. Wenzel, Alex Douglas, Stuart B. Piertney

**Affiliations:** School of Biological Sciences, University of Aberdeen, Aberdeen, United Kingdom; University of Missouri Columbia, UNITED STATES

## Abstract

The increasingly recognised effects of microbiomes on the eco-evolutionary dynamics of their hosts are promoting a view of the “hologenome” as an integral host-symbiont evolutionary entity. For example, sex-ratio distorting reproductive parasites such as *Wolbachia* are well-studied pivotal drivers of invertebrate reproductive processes, and more recent work is highlighting novel effects of microbiome assemblages on host mating behaviour and developmental incompatibilities that underpin or reinforce reproductive isolation processes. However, examining the hologenome and its eco-evolutionary effects in natural populations is challenging because microbiome composition is considerably influenced by environmental factors. Here we illustrate these challenges in a sympatric species complex of intertidal isopods (*Jaera albifrons* spp.) with pervasive sex-ratio distortion and ecological and behavioural reproductive isolation mechanisms. We deep-sequence the bacterial 16S rRNA gene among males and females collected in spring and summer from two coasts in north-east Scotland, and examine microbiome composition with a particular focus on reproductive parasites. Microbiomes of all species were diverse (overall 3,317 unique sequences among 3.8 million reads) and comprised mainly Proteobacteria and Bacteroidetes taxa typical of the marine intertidal zone, in particular *Vibrio* spp. However, we found little evidence of the reproductive parasites *Wolbachia*, *Rickettsia*, *Spiroplasma* and *Cardinium*, suggesting alternative causes of sex-ratio distortion. Notwithstanding, a significant proportion of the variance in microbiome composition among samples was explained by sex (14.1 %), nested within geographic (26.9 %) and seasonal (39.6 %) variance components. The functional relevance of this sex signal was difficult to ascertain given the absence of reproductive parasites, the ephemeral nature of the species assemblages and substantial environmental variability. These results establish the *Jaera albifrons* species complex as an intriguing system for examining the effects of microbiomes on reproductive processes and speciation, and highlight the difficulties associated with snapshot assays of microbiome composition in dynamic and complex environments.

## Introduction

Microbiomes have long been recognised as important functional extensions of their host’s physiological and broader ecological phenotype. For example, microbiomes affect fundamental physiological processes associated with digestion, immune system function, disease aetiology and behaviour [1–3], ecological processes such as nutrient cycling at the plant-root/soil interface [4, 5] and calcification, proliferation and community structure of coral reefs [6, 7], as well as key evolutionary transitions such as the origin of mitochondria [8], gain of photosynthetic function in eukaryotic cells [9] or the parallel and convergent evolution of bioluminescent “light organs” in squid and angler fishes [10, 11]. From an evolutionary perspective, microbiome composition is also implicated in reproductive isolation and speciation via affecting chemosensory cues essential for mating preference [12] or causing fundamental developmental incompatibilities and hybrid breakdown [13]. These insights have given rise to the “hologenome” concept of considering the host macro-organism and its associated microbiome as an integral evolutionary entity [14–16]. As such, studying the multi-layered effects of host-microbiome interactions holds immense value for a broad array of pure and applied disciplines ranging from medicine and agriculture to molecular physiology and ecosystem ecology and evolution [14, 17, 18].

A centrally important phenomenon that underlines how microbiomes may affect eco-evolutionary processes in their hosts is sex-ratio distortion in invertebrates caused by infection with cytoplasmic reproductive endoparasites. *Wolbachia*, *Rickettsia*, *Spiroplasma*, *Cardinium* bacteria and Microsporidian fungi infect the reproductive organs of many arthropod and nematode species and are cytoplasmically transmitted from mother to offspring [19–23]. As a means of promoting transmission and infection prevalence in the population, these parasites manipulate host reproductive biology to distort host sex-ratios in favour of infected females by induction of parthenogenesis, feminization of male offspring, killing of male embryos, disruption of sex-chromosome inheritance, or cytoplasmic incompatibility between individuals with different infection statuses [19, 22, 24]. This demographic disruption can lead to erosion of genetic diversity and phylogenetic signal akin to a bottleneck or selective sweep since most of the population will eventually be descended from few infected matrilines [25, 26]. Conversely, *Wolbachia* infection can also promote diversification via horizontal gene transfer to the host [22], and initiation or reinforcement of reproductive isolation and speciation through cytoplasmic incompatibility between populations with mixed infections [27, 28]. Not least, *Wolbachia* infection can perturb overall microbiome composition, often in sex-specific fashion with downstream physiological effects [29–32]. In concert, these factors firmly establish *Wolbachia* and other reproductive parasites as pivotal agents in driving the evolution of many invertebrates.

Beyond the obvious value in studying prominently important taxa such as *Wolbachia* and other reproductive parasites, key to gaining a proper understanding of the effects of the hologenome on any facet of eco-evolutionary dynamics is the capacity to examine microbiome-wide patterns of diversity in free-living non-model systems. The wide availability of high-throughput DNA sequencing has enabled rapid characterisation of microbial species composition in virtually any type of field sample, and is poised to revolutionise our understanding of how the hologenome operates and evolves in the wild [33, 34]. However, a pre-requisite to thoroughly understanding microbiome composition is an appreciation of potential environmental sources of variation. Abiotic factors in dynamic natural environments may confer considerable spatio-temporal variation in ephemeral uptake and proliferation of commensal microbionts that may not necessarily be functionally linked to host metabolism. For example, microbiome composition in marine copepods is contingent on seasonal and spatial differences in water temperatures [35], and littoral *Hymeniacidon heliophila* sponges can display small-scale variation in microbiome composition between subtidal and intertidal specimens [36]. Conversely, the microbiomes of various marine nematode species are not obviously structured across habitats even on a global scale [37]. These examples highlight the need for an initial assessment of the degree of environmental variation in microbiomes before attempts are made to identify functionally relevant variation in the hologenome and its contribution to host ecology and evolution [38].

The *Jaera albifrons* (sensu lato) species complex of sympatric intertidal isopods is an intuitively attractive study system for examining the links between reproductive parasites, microbiomes and host eco-evolutionary processes. In Europe, the complex comprises *Jaera albifrons* sensu stricto, *Jaera ischiosetosa*, *Jaera praehirsuta* and *Jaera forsmani*, with *Jaera nordmanni* as a congeneric outgroup taxon [39–41]. All species are common across North Atlantic coasts and often form mixed populations in sympatry or parapatry along the intertidal zone. The ingroup species are reproductively isolated through female preference for tactile courtship stimuli administered by males [41, 42], genetic incompatibilities conferring rapid hybrid breakdown [41, 42], and ecological zonation due to species-specific preferences of substrate, drainage, salinity and exposure [41, 43, 44], though some plasticity in the degree of reproductive isolation and frequency of introgressive hybridisation has been noted [41, 45]. In spite of these reproductive isolation mechanisms, all species are polyphyletic according to the mitochondrial 16S rRNA gene and also usually display substantial sex-ratio bias towards females [46–48]. This would be consistent with the presence of sex-ratio distorting reproductive parasites and associated erosion of mitochondrial diversity [42]. Attempts of detecting *Wolbachia* in *Jaera* via PCR have not yielded conclusive evidence for ongoing infection [48, 49]. However, a proper characterisation of the *Jaera* microbiome via next-generation sequencing has not yet been attempted, thus reproductive parasites other than *Wolbachia* may be present and affect *Jaera* demography and evolution. Moreover, such a characterisation would be an invaluable resource for exploring whether the *Jaera* microbiome could be involved in driving speciation and reproductive isolation mechanisms in the species complex, potentially through reinforcement of ecological niche partitioning via metabolic co-adaptation, affecting chemosensory or behavioural mate choice, or developmental hybrid incompatibility [12, 13, 50].

Here we use next-generation amplicon sequencing to provide a first characterisation of the microbiomes of males and females across the *Jaera albifrons* species complex. We deep-sequence the V3/V4 region of the bacterial 16S rRNA gene from DNA pools of *Jaera* individuals, examine specifically whether the reproductive parasites *Wolbachia*, *Rickettsia*, *Spiroplasma* and *Cardinium* are present, and explore broader signatures of seasonal, spatial and sex-specific variation in microbiome composition from samples collected in spring and summer from two coasts in north-east Scotland. This initial description of the *Jaera* microbiome will develop hypotheses for factors affecting microbiome composition in *Jaera* and establish the *Jaera albifrons* species complex as a powerful system for investigating the role of the microbiome in reproductive processes and speciation.

## Materials and methods

### Sample collection and processing

*Jaera* spp. are common intertidal invertebrates that are neither protected nor require sampling permits. Live individuals were collected in spring and summer 2017 from two coasts in north-east Scotland, separated by c. 200 km of coast line. Gardenstown on the north coast (57.672 °N, –2.337 °E) harbours all four European species of the species complex alongside the outgroup *Jaera nordmanni* in varying composition along the shoreline. Two beaches on the south-east coast in close vicinity to each other (Johnshaven: 56.796 °N, –2.328 °E; Arbroath: 56.518 °N, –2.659 °E) harbour >95 % pure single-species populations of *Jaera albifrons* and *Jaera ischiosetosa* respectively.

As soon as possible after collection, individuals were sexed and assigned to species by identifying patterns of pereiopod setation in males using light microscopy [40, 41]. Identified males were then kept at room temperature in a large tub containing filtered sea water from the collection site. Since females cannot be assigned to species morphologically [40, 41], we assigned putative species based on species composition of males collected at the same beach section. An approximately even species mix of females was added to the same tub as the males, and all individuals were starved for one week to reduce gut content. Individuals were then briefly rinsed in sterile water and immediately processed for DNA extraction.

We generated eleven DNA samples that comprised one pure male pool (6-12 individuals) for each of all five species, five mixed or presumably pure female pools (6-12 individuals), and a single exceptionally large female of unknown species (Table 1). These samples not only allowed us to screen males and females of all species for sex-ratio distorting parasites, but also to capture a broad snapshot of the *Jaera* microbiome across coasts and seasons. Samples were homogenized in heated (60 °C) lysis buffer (0.1 M Tris-HCl, 0.05 M EDTA, 0.1 M NaCl, 1 % SDS, 400 μg Proteinase K, 100 μg RNAse A) using an autoclaved teflon plunger. The homogenate was incubated overnight at 60 °C and DNA was extracted via standard phenol-chloroform extraction. DNA quality and quantity were checked with a NanoDrop ND-1000 spectrophotometer. Samples were submitted to Eurofins Genomics (Ebersberg, Germany) for bacterial 16S rRNA V3/V4 amplicon generation (c. 420 bp) using the standard S-D-Bact-0341-b-S-17 and S-D-Bact-0785-a-A-21 primers [51] with sample-specific barcodes, and sequencing on the Illumina MiSeq V3 platform in 300 bp paired-end mode.

**Table 1:** Summary of sequencing effort and sequence diversity across eleven samples.

ID	Species	Season	Region	Sex	Single-end reads	Paired-end reads	Variants	Chao1	ACE	Shannon	Simpson	InvSimpson	Fisher
S1	*Jaera forsmani*	Spring	North	M	355,151	323,324	1,919	2,011 ± 19.433	1,993 ± 21.732	4.938	0.973	37.572	266.718
S2	*Jaera albifrons*	Spring	North	M	285,237	254,668	1,998	2,096 ± 19.66	2,078 ± 22.451	4.719	0.955	22.429	289.874
S3	Mix	Spring	North	F	239,064	218,576	1,409	1,579 ± 40.387	1,544 ± 16.246	5.404	0.984	64.391	198.618
S4	Mix	Spring	South	F	298,546	279,462	1,019	1,342 ± 75.903	1,195 ± 16.267	3.660	0.860	7.127	131.909
S5	*Jaera nordmanni*	Summer	North	M	123,413	75,377	1,344	1,494 ± 29.587	1,469 ± 18.691	5.346	0.985	67.930	210.864
S6	*Jaera ischiosetosa*	Summer	North	M	146,701	95,980	1,344	1,525 ± 35.511	1,474 ± 18.62	4.703	0.946	18.509	204.322
S7	*Jaera praehirsuta*	Summer	North	M	64,878	29,535	1,506	1,603 ± 20.221	1,589 ± 19.683	5.661	0.987	77.825	275.532
S8	Mix	Summer	North	F	537,362	340,550	1,596	1,659 ± 16.183	1,643 ± 19.196	4.716	0.973	36.655	202.424
S9	Unknown	Summer	North	F	776,641	517,273	1,584	1,628 ± 13.248	1,616 ± 18.159	4.389	0.956	22.781	190.543
S10	*Jaera ischiosetosa*	Summer	South	F	480,152	325,520	1,030	1,333 ± 46.439	1,372 ± 19.789	3.957	0.934	15.263	124.762
S11	*Jaera albifrons*	Summer	South	F	559,443	376,747	1,201	1,374 ± 32.229	1,345 ± 18.143	2.896	0.835	6.054	145.490
Total					3,866,588	2,837,012	3,317	–	–	–	–	–	–
Correlation single/paired end (Pearson’s *r*)					–	–	0.952	0.938	0.902	0.992	0.999	0.976	0.964
Association with season (*P*-value)					0.497	0.874	0.240	0.273	0.406	0.766	0.956	0.898	0.515
Association with region (*P*-value)					0.410	0.438	**0.001**	**0.003**	**0.005**	**0.024**	0.084	**0.004**	**0.000**
Association with sex (*P*-value)					**0.002**	**0.021**	**0.045**	0.118	0.120	0.057	0.137	0.232	**0.006**

Sample descriptors (species, season, region and sex) are given alongside numbers of de-noised single-end and paired-end reads, and the following diversity indices based on single-end reads: numbers of unique sequence variants, Chao1 ± SE, ACE ± SE, Shannon, Simpson, inverse Simpson and Fisher index. Below, Pearson’s correlation coefficient (*r*; all *P* ≪ 0.001) between single-end and paired-end datasets, and associations of metrics with sample descriptors (two-tailed Welch’s *t*-test *P*-value) are presented. Significant *P*-values (*P* ≤ 0.05) are emboldened.

### Sequence assembly, curation and taxonomic classification

Raw sequence reads were filtered, de-noised and assembled to unique single-end (forward reads only) as well as paired-end sequence variants using dada2 v1.4.0 [52] in r v3.4.0 [53]. Reads were trimmed at nucleotide call quality below 2, and reads with undetermined bases were discarded. Exploratory quality plots indicated a rapid decline in basecall quality towards the ends of the reads, particularly for reverse reads. Therefore, for paired-end analysis, forward reads were further trimmed to 260 bp and reverse reads to 220 bp, ensuring an overlap of at least 60 bp. Error rates were estimated and sequences were de-noised separately for forward and reverse reads in pooled-sample mode. Single-end and paired-end contigs were assembled from de-noised data and chimera sequences were removed.

Taxonomic classification to genus level was assigned from the silva nr v128 database [54] using the RDP classifier algorithm [55] with *k*-mer size 8, 100 bootstrap replicates and a minimum bootstrap support of 50. Species-level classification was added where possible based on 100 % sequence identity with silva nr v128 [56]. Sequences that were assigned to chloroplast, mitochondria, archaea, or eukaryota taxa were removed. Each sample was further annotated with putative functional metabolic capabilities of the identified microbial community using tax4fun v0.3.1 and associated pre-computed silva nr v123 reference data [57]. The observed sequence counts were transformed into abundances of KEGG enzymes via association with KEGG reference organisms. These enzymes were further classified with the first three levels in KEGG functional hierarchies [58].

The taxonomically classified microbiomes were then screened for reproductive parasite species in the *Wolbachia*, *Rickettsia*, *Spiroplasma* and *Cardinium* genera or relevant higher taxonomic levels. Candidate sequences were aligned with all available *Wolbachia*, *Rickettsia*, *Spiroplasma* and *Cardinium* reference sequences in silva nr v128 using mafft v7.305 [59] and clustered using neighbour-joining on Kimura-2-parameter phylogenetic distances in ape v4.1 [60]. Sequences that clustered closely with the silva reference sequences were more closely examined using ncbi megablast [61] against the non-redundant nucleotide collection (nt).

### Microbiome sequence diversity and composition

Sequence diversity analyses were carried out on single-end as well as paired-end datasets, using r and the package phyloseq v1.19.1 [62]. Sequencing depths per sample were examined for circumstantial associations with the categorical sample variables season (spring and summer), region (north: Gardenstown; south: Johnshaven/Arbroath) and sex using negative binomial generalized linear models (GLM) in the mass package [63]. Rarefaction curves were obtained by computing the number of unique sequence variants in subsamples of increasing sizes in steps of 1,000 sequences without replacement [64]. Diversity indices (Chao1, ACE, Shannon, Simpson, inverse Simpson and Fisher) were computed for each sample and compared between samples grouped by season, region or sex using two-tailed Welch’s *t*-test. Consistency of all metrics between single-end and paired-end datasets was examined using Pearson’s correlation test.

Rarefied versions of the datasets were obtained by subsampling to the lowest sequencing depth across samples. Microbiome structure between samples was explored with Jaccard and Bray-Curtis dissimilarity indices and visualised in two-dimensional space using metric (Jaccard) or non-metric (Bray-Curtis) multidimensional scaling (MDS). Samples were then clustered hierarchically using Ward’s criterion on the dissimilarity matrix, and clusters were visualised as dendrograms using ggtree v1.6.10 [65]. Sources of variation attributed to season, region and sex were explored with distance-based redundancy analysis and permutational multivariate analysis of variance (PERMANOVA) with 9,999 permutations [66]. Finally, to further explore sex-specific differences in microbiome composition, we fitted negative binomial GLMs in a differential gene expression framework that accounts for differences in library size and dispersion, as implemented in deseq2 [67]. Fold changes were calculated between sexes accounting for season and region as covariates. *P*-values were corrected for multiple testing using the false-discovery rate method [68], and sequences with significant fold changes (FDR ≤ 0.1) were identified.

## Results

### Taxonomic composition and diversity

De-noised single-end sequence data comprised 64,878–776,641 reads that collapsed to 1,019–1,998 unique sequence variants per sample. Across all eleven samples, 3,317 unique sequence variants were observed, which were assigned to 25 phyla, 45 classes, 94 orders, 185 families and 445 genera, based on the silva nr v128 database. However, considerable fractions of these sequence variants could not be assigned beyond particular taxon levels at the 50 % bootstrap cut-off, i.e., 0.78 % for phylum, 3.33 % for class, 8.06 % for order, 16.21 % for family, 42.15 % for genus and 94.16 % for species levels. Paired-end data recovered less diversity, comprising 29,535–517,273 reads and 329–1,800 unique sequence variants, and captured less diversity with 3,283 unique sequence variants assigned to 23 phyla, 40 classes, 89 orders, 182 families and 426 genera. However, taxonomy assignment was slightly better compared to single-end data, with non-classification rates of 0.45 % for phylum, 1.58 % for class, 4.28 % for order, 10.70 % for family, 35.93 % for genus and 93.97 % for species. Rarefaction curves approached asymptotic stages for most samples, suggesting that the sequencing effort captured the majority of sequence diversity in both types of datasets (S1 Fig).

**Fig 1:**
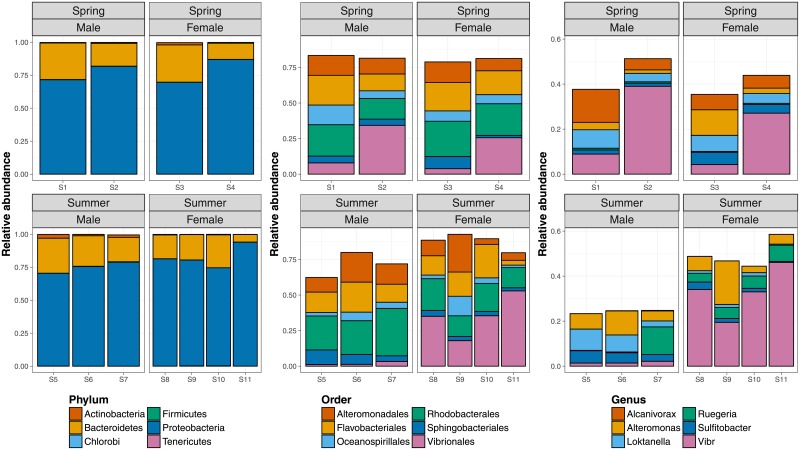
Relative sequence abundances of the six most abundant phyla, orders and genera across eleven samples (S1-S11), organised by season and sex.

The Proteobacteria and Bacteroidetes were the dominant phyla in all samples, accounting for 69.7–94.0 % and 5.7–28.2 % of sequences per sample, and the six most abundant phyla accounted for 99.5–99.9 % (Fig 1). The six most abundant orders accounted for 62.4–92.7 % and the six most abundant genera for 23.3–58.6 % of sequences, of which *Vibrio* dominated most samples with up to 46.1 %. Notwithstanding, microbial sequence diversity was high in all samples, with a Simpson index of 0.835–0.987 and Fisher index of 125–290 (Table 1; S2 Fig). All metrics were highly correlated between single-end and paired-end datasets (*r* = 0.902 − 0.999;*p* ≪ 0.001; Table 1). Diversity was similar among seasons, but signatures of region and, in particular, sex were apparent in many diversity metrics (S2 Fig). Rigorous statistical analysis beyond basic Welch’s *t*-tests was precluded by low sample size, but these tests supported a difference between sexes in particular (Table 1; S2 Fig), consistent with shallower rarefaction curves in females (S1 Fig).

Prediction of broad-brush metabolic capacity of the identified microbial communities via sequence similarity of taxonomically classified sequences to KEGG model taxa recovered 278 KEGG pathways. The proportion of sequences that could not be mapped to KEGG organisms (FTU) ranged from 55.0 % to 96.8 % (median 89.2 %) per sample. The most abundant top-level category was 09100 Metabolism (median 57.3 % across samples), followed by 09130 Environmental Information Processing (23.9 %). The most abundant pathways included 09131 Membrane transport, 09102 Energy metabolism and 09101 carbohydrate metabolism (S3 Fig).

### Sex-ratio distorting reproductive parasites

**Fig 2:**
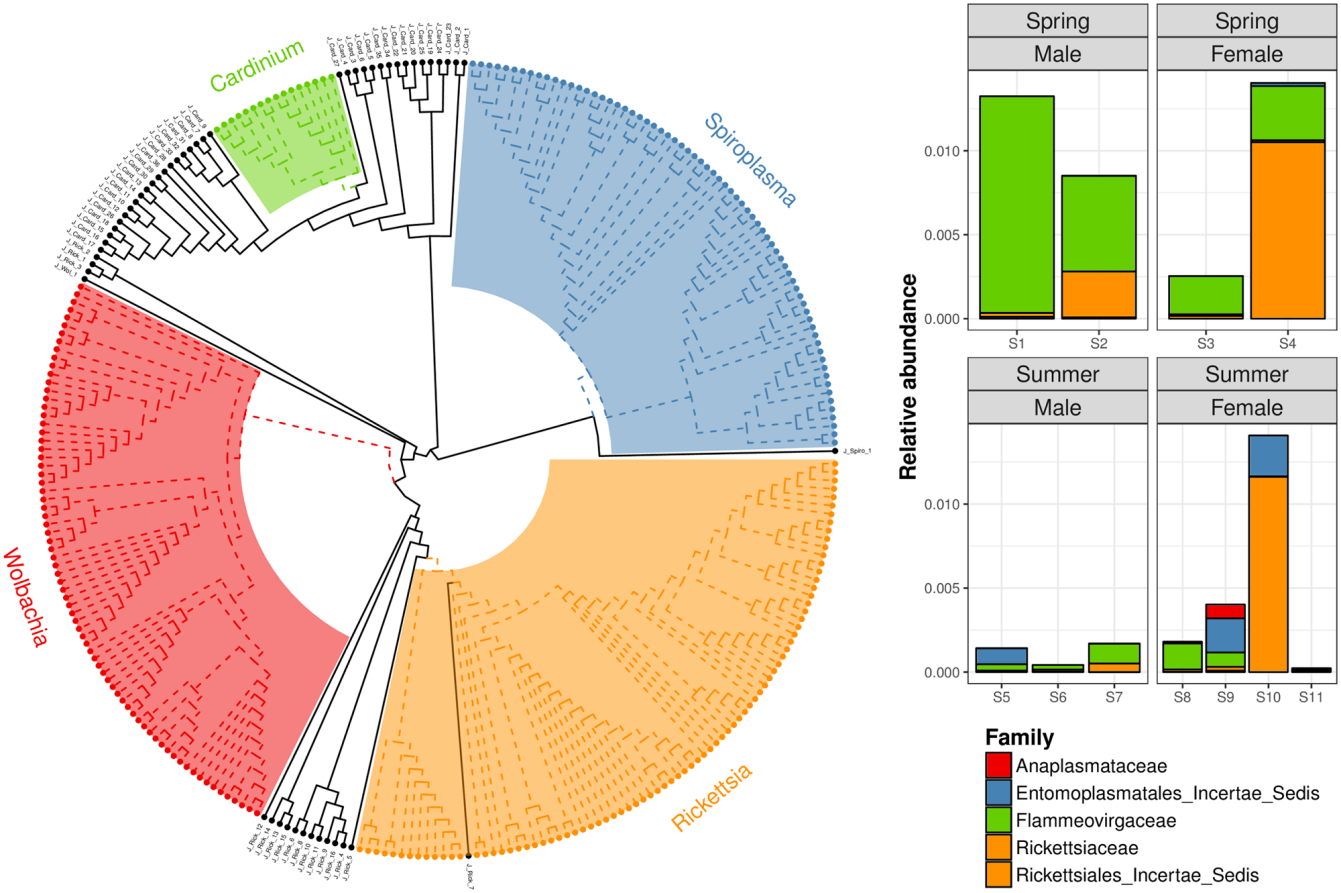
Neighbour-joining dendrogram (K2P phylogenetic distance) of SILVA NR v128 16S rRNA gene reference sequences for reproductive parasites *Wolbachia*, *Rickettsia*, *Spiroplasma* and *Cardinium* genera (dashed branches) and most closely related *Jaera* 16S rRNA gene sequences (families Rickettsiaceae and Flammeovirgaceae, and order Entomoplasmatales; solid branches). The relative sequence abundances of the *Jaera* sequences are summarised alongside. Tip labels correspond to sequence identifiers in S1 Table.

No sequences were directly assigned to known silva strains of the reproductive parasite genera *Wolbachia*, *Rickettsia*, *Spiroplasma* and *Cardinium*. However, some sequences were assigned to relevant higher taxonomic ranks: one sequence to the family Anaplasmatacea, 16 sequences to Rickettsiacea, 36 sequences to Flammeovirgaceae and one sequence to the order Entomoplasmatales. The counts of these sequences ranged from 0 to 5,583, representing relative abundances of at best 1.16 % (Fig 2; S1 Table). Of these 54 sequences, three clustered reasonably closely with the *Rickettsia*, *Spiroplasma* and *Cardinium* clades, but no sequence clustered closely with *Wolbachia* (Fig 2).Megablast broadly supported these classifications, matching an uncultured Rickettsiaceae bacterium (accession JQ701668.1) at 90 % identity to the *Rickettsia* sequence, and an uncultured bacterium from the Cytophaga-Flavobacterium-Bacteroides group (DQ812543.1) at 98 % identity to the *Cardinium* sequence. However, the presumed *Spiroplasma* sequence matched with 94 % identity an uncultured bacterium from the Mycoplasmataceae family (EU646196.1), which is situated in a different order than *Spiroplasma*.

### Sources of variation in microbiome composition

We further explored microbiome composition for sex-specific, seasonal and regional signatures. Since sequencing depth was different between male and female samples (negative binomial GLM: *z* = 3.115;*P* = 0.002), the datasets were rarefied (64,878 sequences for single-end data and 29,535 sequences for paired-end data) to avoid spurious sex-specific signatures in microbiome composition.

**Fig 3:**
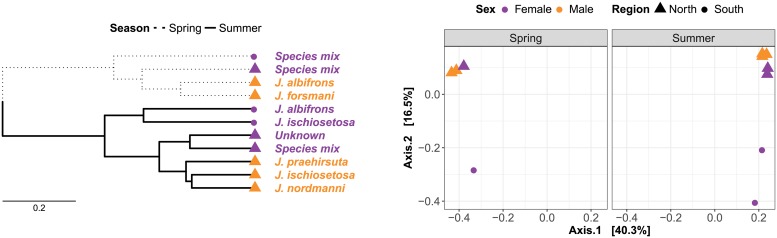
Hierarchical clustering (left) and metric multidimensional scaling (right) of Jaccard dissimilarity among samples. Sample categories (season, region and sex) are indicated by line type, symbol shape and colour, respectively.

Ordination and hierarchical clustering of Jaccard dissimilarity among samples suggested two major clusters that correspond to samples collected in spring and summer. Within both seasons, samples are further clustered by geographic region, and the northern region is further subdivided by sex (Fig 3). Distance-based redundancy analysis ascribed 39.6 % of the total variance to season, 26.9 % to region and 14.1 % to sex, and all three hierarchical variance components were statistically significant (Table 2). Bray-Curtis dissimilarity broadly supported these patterns (Table 2), but hierarchical clustering did not consistently recover the same nested structure (S4 Fig).

**Table 2:** Permutational multivariate analysis of variance (PERMANOVA) in Jaccard and Bray-Curtis dissimilarity indices among samples.

	DF	SS	MS	*F*	*R*^2^	*P*
*Jaccard dissimilarity*						
Season	1	0.931	0.931	10.141	0.396	**0.000**
Region	2	0.633	0.316	3.445	0.269	**0.002**
Sex	2	0.331	0.166	1.803	0.141	**0.049**
Residuals	5	0.459	0.092	0.195	–	–
Total	10	2.354	1.000	–	–	–
*Bray-Curtis dissimilarity*						
Season	1	0.704	0.704	4.388	0.245	**0.000**
Region	2	0.772	0.386	2.407	0.268	**0.001**
Sex	2	0.599	0.299	1.866	0.208	**0.018**
Residuals	5	0.802	0.160	0.279	–	–
Total	10	2.876	1.000	–	–	–

Total variance was decomposed into hierarchical levels corresponding to season, region and sex, and statistical significance was estimated from 9,999 permutations. The table presents degrees of freedom (DF), sums of squares (SS), mean squares (MS), *F*-statistic, *R*-squared and *P*-value. Significant *P*-values (*P* ≤ 0.05) are emboldened.

Since all samples from the southern regions were females, it cannot be ruled out that variance ascribed to region is in fact sex-specific variation. However, this is quite unlikely since variance among males and females in the northern region in summer was considerably smaller than the putative variance among regions in summer (Fig 3). Similarly, although not all species are represented within each cluster, it appears that structure among sexes outweighs structure associated with species or microgeography at the same beach. *Jaera nordmanni* and *Jaera ischiosetosa* males collected from the same set of rocks in Gardenstown (north) in summer clustered most closely, followed by *Jaera praehirsuta* males collected further down the shore at the same time. However, the single large female (unknown species) collected at the same beach and time did not cluster as closely with any of these three male single-species samples. Instead, she clustered most closely with a mix of females collected from the same rocks as the three male samples (Fig 3).

**Fig 4:**
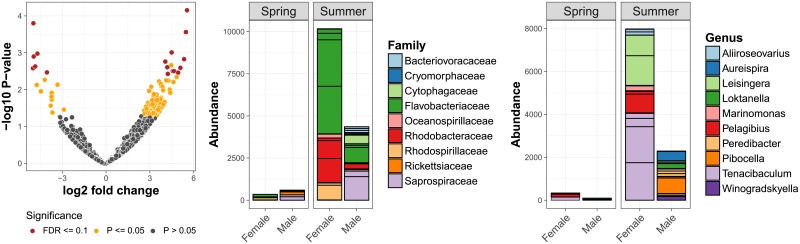
Sequence variants with differential abundance between sexes. The left panel summarises fold change and statistical significance for each sequence variant. The following two panels illustrate total aggregated sequence counts (abundance) and taxonomic classification (family or genus) of statistically significant (FDR ≤ 0.1) sequence variants.

Exploring the identified sex-specific signal in microbiome composition further with negative binomial models indicated that eleven sequences were significantly (FDR ≤ 0.1) more abundant in males and six sequences were more abundant in females, after accounting for differences in season and region (Fig 4). Of these 17 sequences ten had taxonomic annotation, representing ten genera in nine families: *Aureispira*, *Peredibacter*, *Loktanella*, *Winogradskyella* and *Pibocella* genera were more abundant in males, and *Tenacibaculum*, *Marinomonas*, *Aliiroseovarius*, *Leisingera* and *Pelagibius* genera were more abundant in females (Fig 4). A simplified analysis within the northern region only recovered similar patterns, corroborating *Pibocella* as male-associated and suggesting *Vibrio* and *Owenweeksia* as additional female-associated taxa (S5 Fig). Likewise, using non-rarefied data and a stricter significance threshold, a similar set of differentially abundant taxa was identified, highlighting *Aliivibrio*, *Flavirhabdus* and *Polaribacter* as further female-associated taxa (S6 Fig).

## Discussion

We present an initial survey of microbiome composition among males and females of all UK members of the *Jaera albifrons* species complex of intertidal isopods. The salient features of all microbiomes are high species diversity and absence of the classic feminizing reproductive parasite *Wolbachia*, though potentially novel strains of *Rickettsia* and *Cardinium* may be present instead. Additionally, microbiome composition varied considerably among samples and revealed hierarchical structure associated with season, region and sex. These patterns provide a first look at environmental sources of variation in microbial assemblages and could indicate an involvement of the microbiome in reproductive processes in *Jaera*.

### Characterisation of *Jaera* microbiomes

The microbial communities of all samples were dominated by the Proteobacteria and Bacteroidetes phyla, which are widely described as the most abundant phyla in intertidal and open oceanic environments [36, 69–72]. The high abundance of *Vibrio* in particular is consistent with microbiomes of other marine invertebrates such as copepods [35] or sea urchins [73]. *Vibrio* is a very common endo- and epibiont in marine crustaceans and often produces chitinolytic enzymes that allow for exploiting chitinous exoskeleton as a niche for attachment and proliferation [71, 74, 75]. Some *Vibrio* species are pathogens and others have been implicated in biogeochemical processes, but the specific metabolic relationships between crustaceans and *Vibrio* are cryptic [74].

Beyond these dominant taxa, sequence diversity in *Jaera* was high, consistent with diversity in intertidal sponges [36] and other marine invertebrates [35], and exceeded diversity of the terrestrial isopod *Armadillidium vulgare* [31]. A large proportion of sequences could not be taxonomically characterised to species level, and functional classification was hampered by very low mapping rate of sequences to KEGG organisms. Rarefaction curves suggested that more sequencing effort would have detected even more diversity in most samples, particularly in males. This would suggest a wealth of uncharacterised taxonomic diversity, consistent with other studies investigating marine microbiomes [69, 76], and highlights the need for better reference characterisation of marine microbial communities [34].

In spite of capturing high microbial diversity, there was no evidence of known silva-curated strains of the reproductive parasites *Wolbachia*, *Rickettsia*, *Spiroplasma* and *Cardinium*. Although some sequences were classified to relevant higher taxonomic ranks and clustered relatively closely with known silva strains, only one sequence (presumed *Rickettsia*) formed a monophyletic group with known strains. Even if some of the identified sequences represented novel, somewhat diverged, strains of these reproductive parasites, all sequences in question had very low abundances and would not suggest high infection intensities. These results are difficult to reconcile with prevalent sex-ratio distortion in *Jaera* [46–48] and pervasive *Wolbachia* infection in many crustaceans [49, 77]. However, these results are fully compatible with previous studies that have failed to reliably detect *Wolbachia* infection in total *Jaera* DNA extracts using targeted PCR assays [48, 49]. Ribardière et al. [48] screened 817 individuals across the *Jaera albifrons* species complex using 11 PCR protocols, but found little evidence of infection beyond an ephemeral novel haplotype in some *Jaera albifrons* and *Jaera praehirsuta* individuals, identified using a nested PCR protocol. As such, infection of *Jaera* species by *Wolbachia* or other bacterial sex-ratio distorting parasites cannot be ruled out, but infection intensities and prevalence appear to be very low and difficult to detect.

The biological relevance of rare sequences is difficult to assess and may well be an artefact of working with whole-body DNA extracts. Reproductive parasites primarily infect the reproductive and digestive tracts, thus it may be possible that dissection of these tissues prior to DNA extraction and sequencing improves detection [31, 78, 79]. Nevertheless, whole-body extracts should not in principle preclude detection of *Wolbachia* infection [29, 80], and even low infection levels should be readily detectable [81]. We thus conclude that prokaryotic reproductive parasites are unlikely to explain pervasive sex-ratio biases among the *Jaera albifrons* species complex, but note that the role of eukaryotic sex-ratio distorters (such as Microsporidian fungi [23]) remains uninvestigated in *Jaera*.

### Sources of variation in microbiome composition

Microbiome composition in *Jaera* varied considerably between spring and summer despite similar species richness, suggesting that the microbiome in *Jaera* undergoes extensive temporal changes in species composition, yet remains fairly consistent in complexity. Seasonal changes in marine microbiomes are well-documented and are primarily driven by abiotic environmental factors such as temperature and biogeochemical processes [69, 82, 83]. In copepods, such seasonal changes have been reported even across a few weeks in early summer and may be linked to a rise in water temperatures and concomitant changes in temperature-sensitive marine microbial communities [35]. Any vacated ecological niches would then be taken up by different microbe species such that the overall species richness would not be greatly affected. For example, *Vibrio* form the core microbiome of copepods in subtropical locations, but *Vibrio* abundance is lower in temperate regions where a similar chitinolytic niche could be taken up by *Pseudoalteromonas* species [35].

The *Jaera* microbiome also showed regional structure in species richness and species composition across the two sampling regions in north-east Scotland. Although we sampled only females from the two southern coasts and therefore cannot rule out that at least part of the regional variance is attributable to species rather than geography, spatial effects on littoral and intertidal microbiome composition are, in fact, commonly reported at large and small scales. This is illustrated by vastly different microbiomes in *Hymeniacidon heliophila* sponges in subtidal and intertidal habitats at the same site [36], as well as spatial structure in microbiomes in populations of sand hoppers across the Tuscan coast [70] and benthic amphipods in the Great Lakes [84]. The regional structure in the *Jaera* microbiome could be due to differences in abiotic factors such as aspect (north-facing versus east-facing beaches), geology (cliff coast vs. plain coast) and human activity (proximity to harbours or sewage outlets). One particular factor could be differences in salinity, either intrinsic to the substrate or extrinsic from nearby freshwater streams. Both beaches in the south were affected by freshwater drainage from streams, whereas the beach in the north had no freshwater influx. The observed lower microbial diversity in the freshwater-affected sites contradicts the pattern found in a freshwater-marine transect in Greece [76], but is consistent with an increase in species richness and considerable changes in composition of the skin microbiome of Atlantic salmon after transitioning from freshwater to seawater [85].

Finally, overall microbiome composition showed a signature of sex, nested within the larger environmental variance components. Males tended to have more diverse microbiomes than females and the sexes also differed in the presence and abundance of a range of taxa, including a range of putative pathogens such as *Aliivibrio*, *Aliiroseovarius*, *Vibrio* and *Tenacibaculum* [86–88], and common environmental species typical of marine arthropods with no immediate functional link to reproductive processes, such as *Loktanella*, *Glaciecola*, *Aureispira*, *Winogradskyella*, *Pelagibius* and *Marinomonas* [75, 89]. An interesting finding was the high abundance of *Leisingera* in females in summer, alongside *Vibrio* and *Tenacibaculum*. Secondary metabolites produced by *Leisingera* are known to have antimicrobial effects and are used by cephalopods to protect their eggs against pathogens such as *Vibrio* [90]. Nevertheless, the specific functional roles of the taxa assemblage in male and female *Jaera* remains obscure and will require more detailed functional assays and experimentation.

Sex-specific differences in diversity and composition have been reported, for example, in whole-body microbiomes of phloem-feeding whiteflies, aphids and psyllids [29], and cloacal microbiomes of the striped plateau lizard *Sceloporus virgatus* [91]. In arthropods, these differences are often attributed to infection with reproductive endoparasites [29, 32]. For example, uninfected males and females of the terrestrial isopod *Armadillidium vulgare* have similar microbiomes, but *Wolbachia*-infected females carry higher total bacterial loads [30, 31]. Since we found little evidence of reproductive parasites in *Jaera*, alternative explanations need to be considered. One hypothesis could be that sex-specific microbiomes are functionally linked to intra-specific reproductive processes such as sex recognition or sexual selection that may have knock-on effects on reproductive isolation and speciation [12, 13, 92]. For example, reproductive isolation via pheromones is documented in allopatric populations of the marine polychaete *Neanthes acuminata* [93] and sympatric populations of the amphipod *Eogammarus confervicolus* [50]. The idea that microbiomes may be linked to host developmental processes and co-diverge tightly with host speciation events—a phenomenon termed “phylosymbiosis”—is a hotly debated topic [14–16, 38]. Although we were unable to separate species-specific signals from sex-specific signals with the present set of samples, the *Jaera albifrons* species complex would be an excellent study system for testing these ideas with more extensive sampling across both sexes within all species.

Conversely, instead of enhancing metabolic function or causing behavioural changes in the host, sex-specific patterns in microbiont abundance could simply be attributed to differences in host body size or behaviour that circumstantially cause differential uptake and proliferation of episymbiont communities. Male *Jaera* are usually smaller than females despite evidence of sexual selection for body size and size-assortative mating [40, 94], suggesting that the effect of body size on microbiome composition would be worth investigating further [95]. Male *Jaera* could also potentially occupy different microhabitats than females as a consequence of sexual dimorphism [96]. Although all samples were collected from underneath rocks, which always harbour mixed sex populations [46, 47], sex-specific differences in substrate microhabitat occupation cannot be ruled out but are yet to be investigated. Similarly, behavioural differences could also affect both epi- and endosymbiont communities, for example through differences in feeding rates or food preferences among sexes, which is well supported in copepods [97]. Gravid females in particular would be expected to change feeding habits or even cease feeding altogether, as is the case in the amphipod *Ligia* [98]. All sampled *Jaera* females were not gravid, but sexual receptivity or other ongoing reproductive processes may well have caused sex differences among microbiomes, particularly during the reproductive peak in summer where these differences were most pronounced [46].

### Outlook and conclusions

In summary, the *Jaera* microbiome is highly diverse and appears to be subject to multiple spatio-temporal environmental sources of variation, which is typical of marine intertidal microbiomes. A surprising result was the weak evidence of sex-ratio distorting reproductive parasites, which suggested very low infection levels at best in spite of pervasive sex-ratio distortion. However, the finding of sex-specific patterns in overall microbiome composition warrants closer scrutiny and establishes the *Jaera albifrons* species complex as an intriguing study system for the effects of microbiomes on host reproductive processes.

Our study has provided a snapshot assay that highlights the vast amount of variation in microbiomes from highly dynamic and complex environments. No doubt much of the ephemeral variation that has been characterised is attributed to ephemeral epibionts that may not necessarily be linked to host metabolism. Variation in this fraction could be reduced by maintaining *Jaera* long-term under controlled common-garden conditions [38]. Similarly, dissecting digestive or reproductive tracts could be a worthwhile avenue for targeting more specialised symbionts, since these tissues are often dominated by few taxa in tight association with host metabolism [73, 99–101]. Notwithstanding, our study highlights that, in order to properly understand the causes and consequences of phylosymbiosis or other effects of the hologenome it is essential to characterise microbiomes *in situ* with an appropriate sampling design that allows for appreciation of all sources of extrinsic and intrinsic variation. As such, more descriptive studies are essential for generating hypotheses of how the hologenome may operate in complex environments beyond classic model systems or controlled laboratory environments [34].

## Supporting information

S1 Fig Rarefaction curves for male and female samples in single-end and paired-end assemblies. (PDF)

S2 Fig Microbial diversity metrics in samples grouped by season, region and sex. (PDF)

S3 Fig Median relative abundance of predicted KEGG pathways. (PDF)

S4 Fig Hierarchical clustering and multidimensional scaling of Jaccard and Bray-Curtis dissimilarities in single-end and paired-end assemblies. (PDF)

S5 Fig Fold changes and taxonomic classification of sequence variants with differential abundance between sexes (northern region only), based on rarefied data. The top panels represent the full dataset of eleven samples; the bottom panels represent the eight samples from the northern region only.
(PDF)

S6 Fig Fold changes and taxonomic classification of sequence variants with differential abundance between sexes, based on non-rarefied data. The top panels represent the full dataset of eleven samples; the bottom panels represent the eight samples from the northern region only.
(PDF)

S1 Table Total read counts and relative abundances of sequences closely related to SILVA-strains of reproductive parasites *Wolbachia*, *Rickettsia*, *Spiroplasma* and *Cardinium*. (XLSX)
