# Supplementary figures and images for: Microbiome composition within a sympatric species complex of intertidal isopods (Jaera albifrons)
Source: PLoS One. 2018 Aug 29;13(8):e0202212. doi: 10.1371/journal.pone.0202212 (PMC6114722; doi:10.1371/journal.pone.0202212)

Single-end

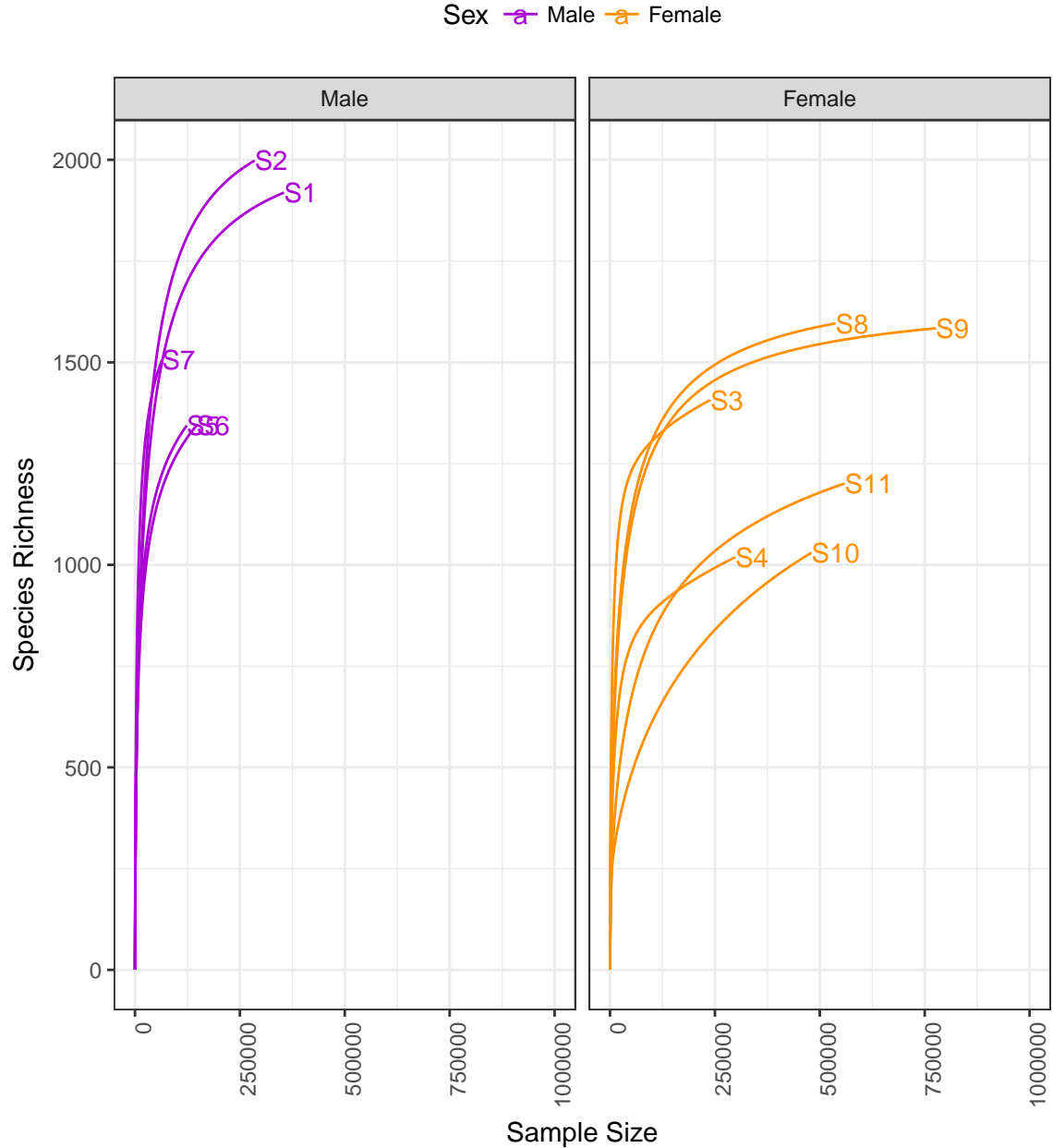

Paired-end

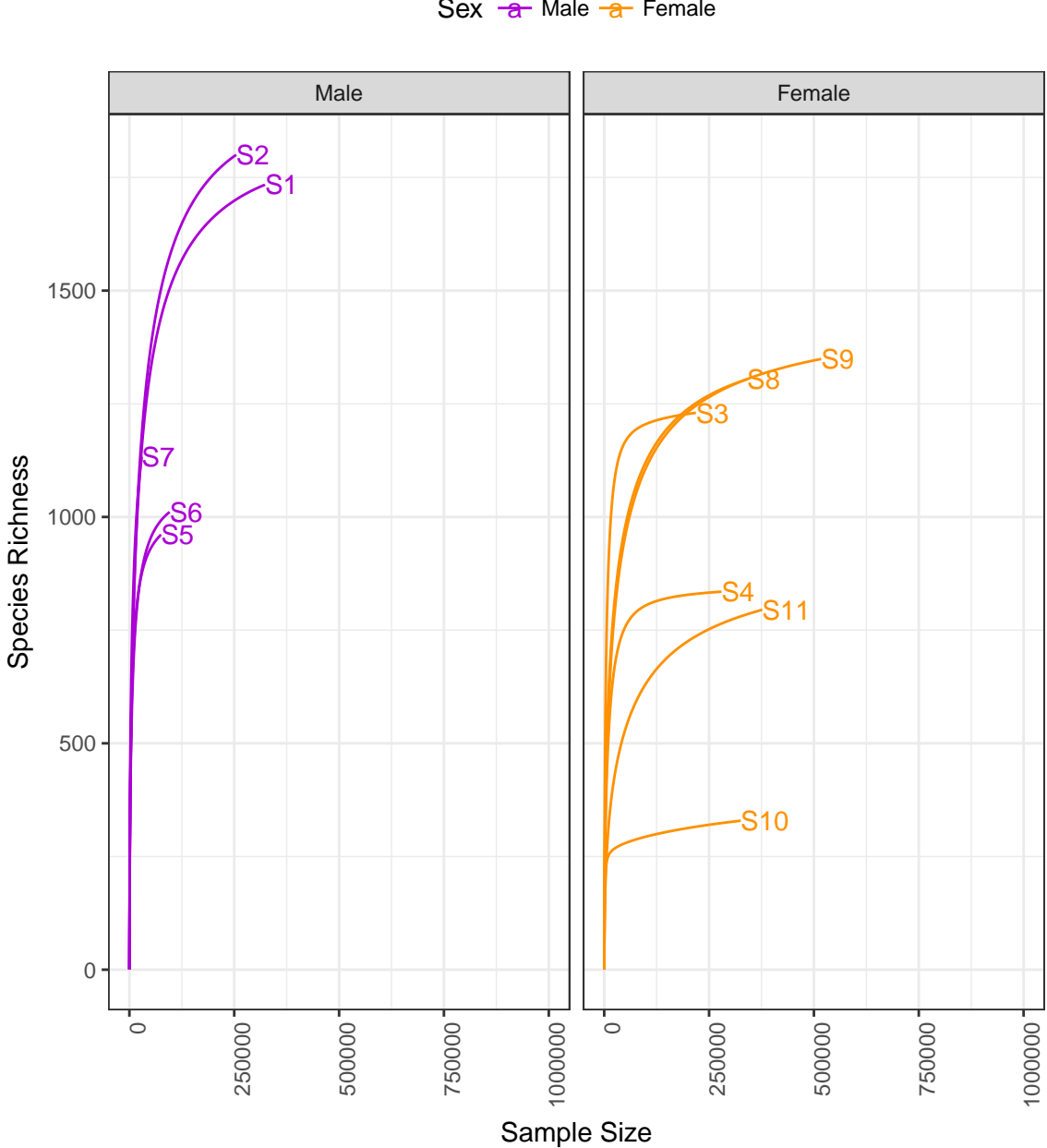

Supplement: S1 Fig — (PDF) [file pone.0202212.s001.pdf]

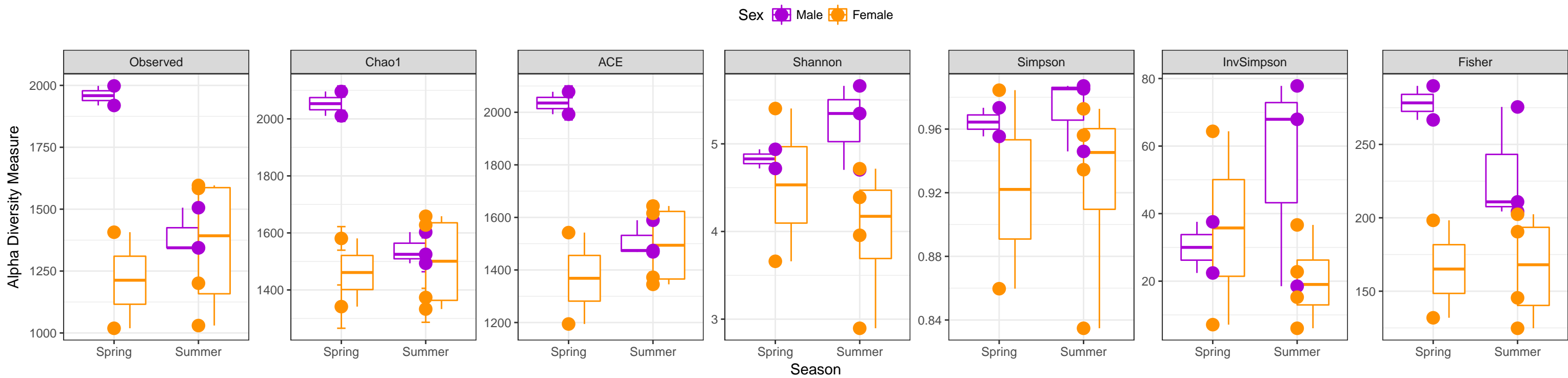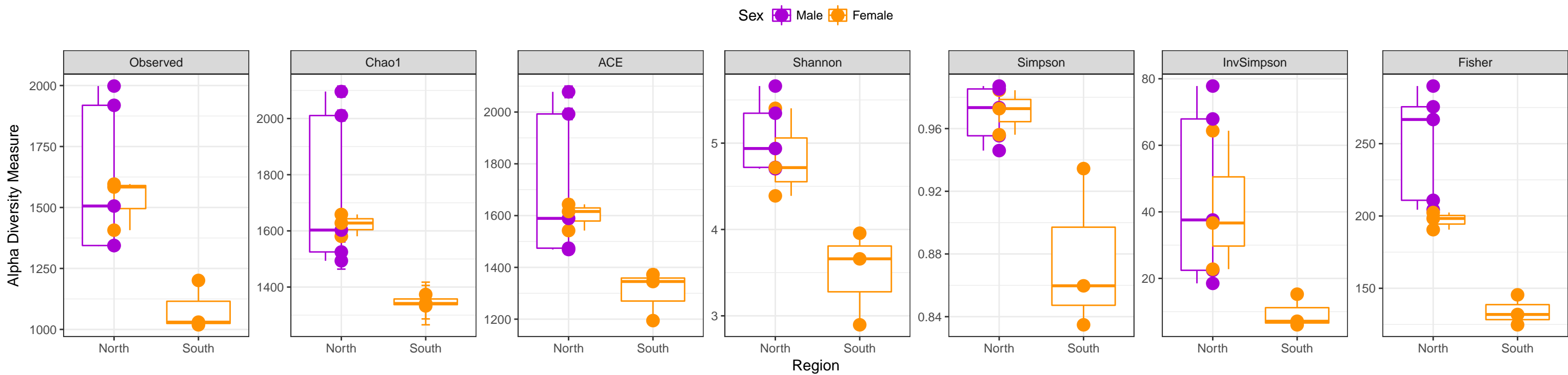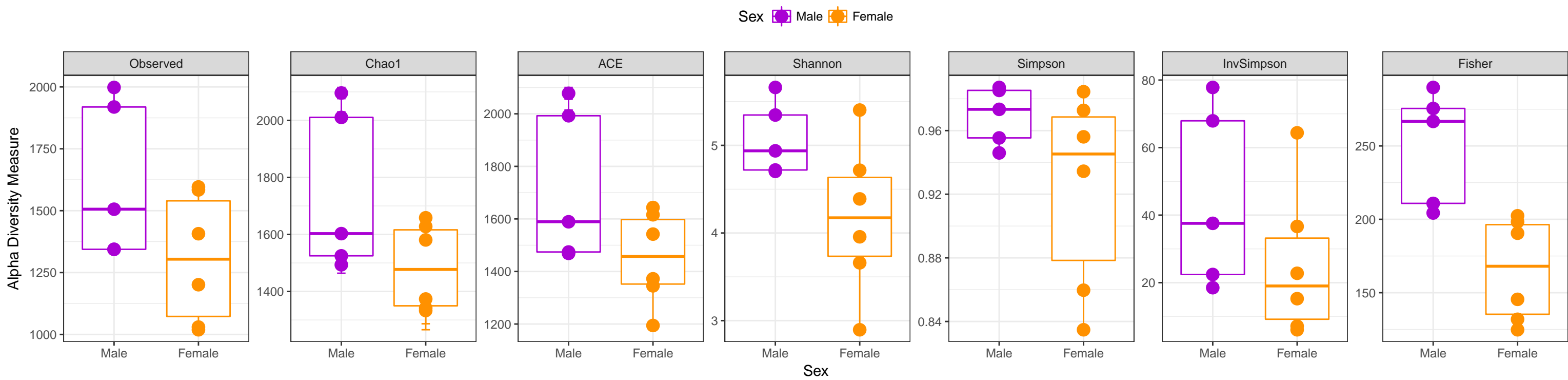

Supplement: S2 Fig — (PDF) [file pone.0202212.s002.pdf]

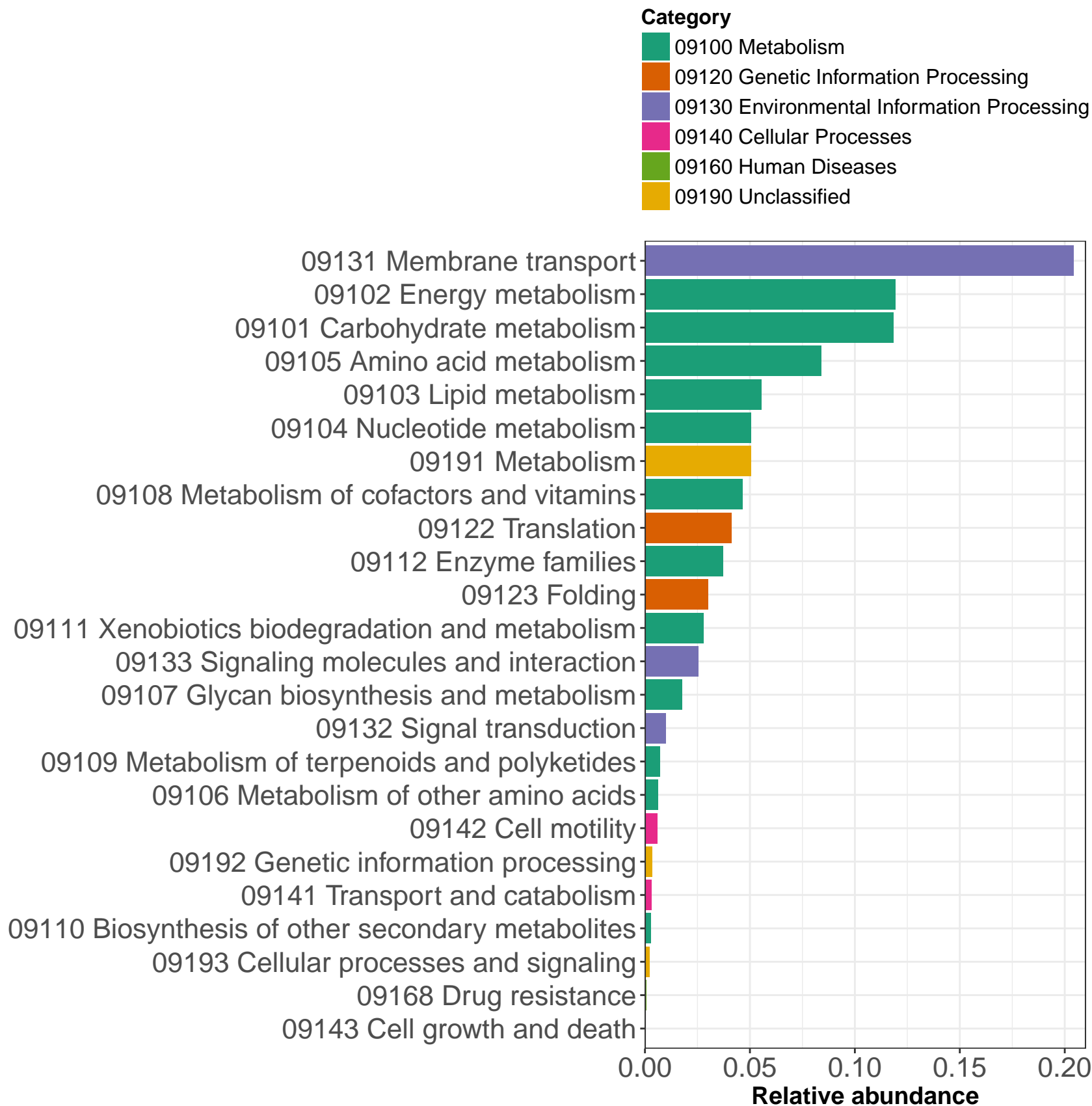

Supplement: S3 Fig — (PDF) [file pone.0202212.s003.pdf]

Jaccard single-end

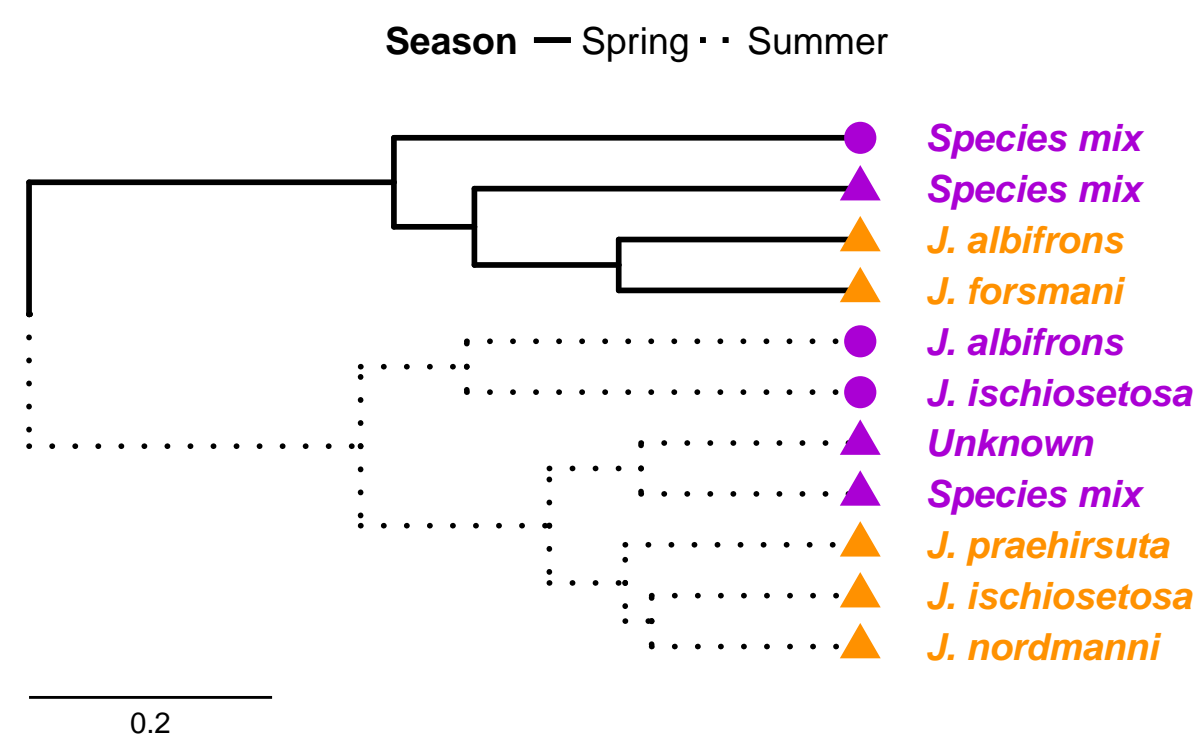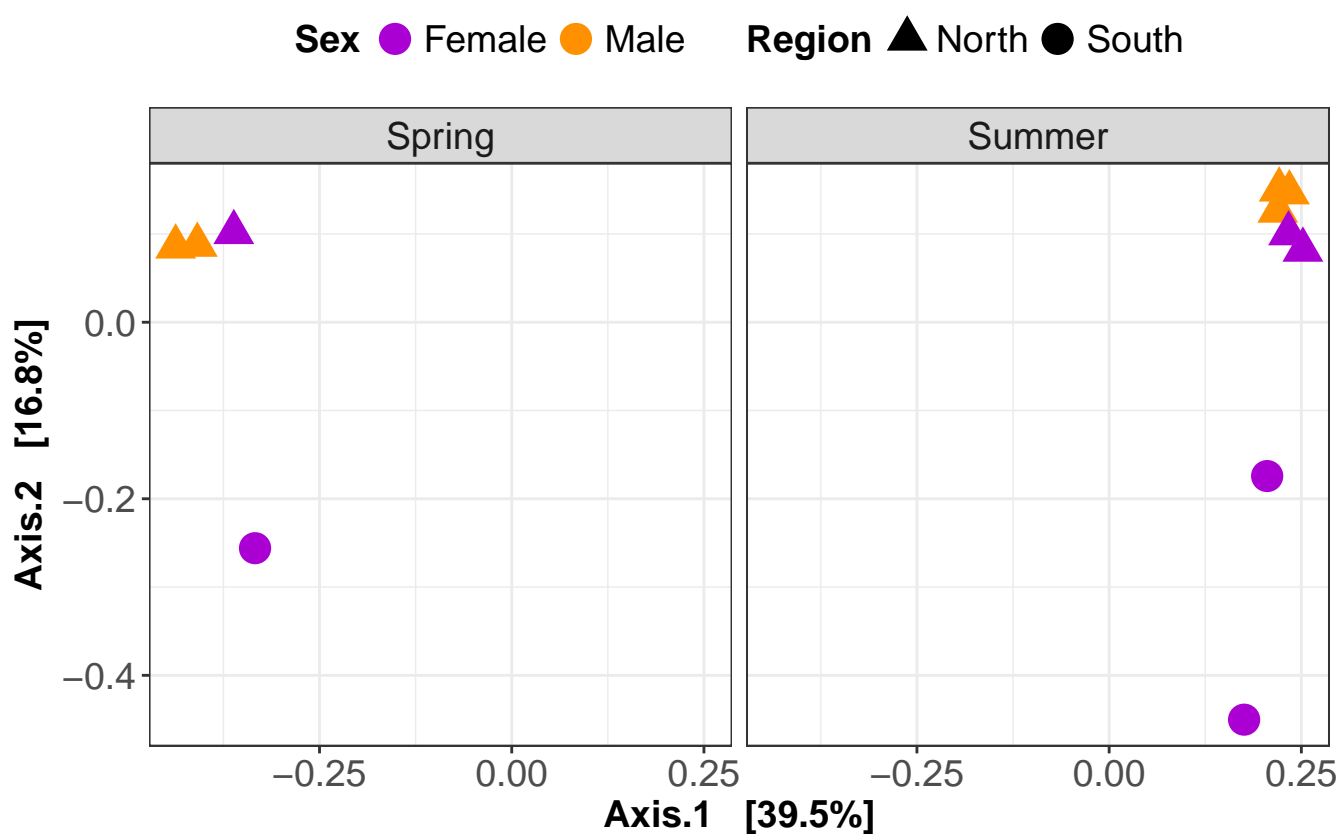

Jaccard paired-end

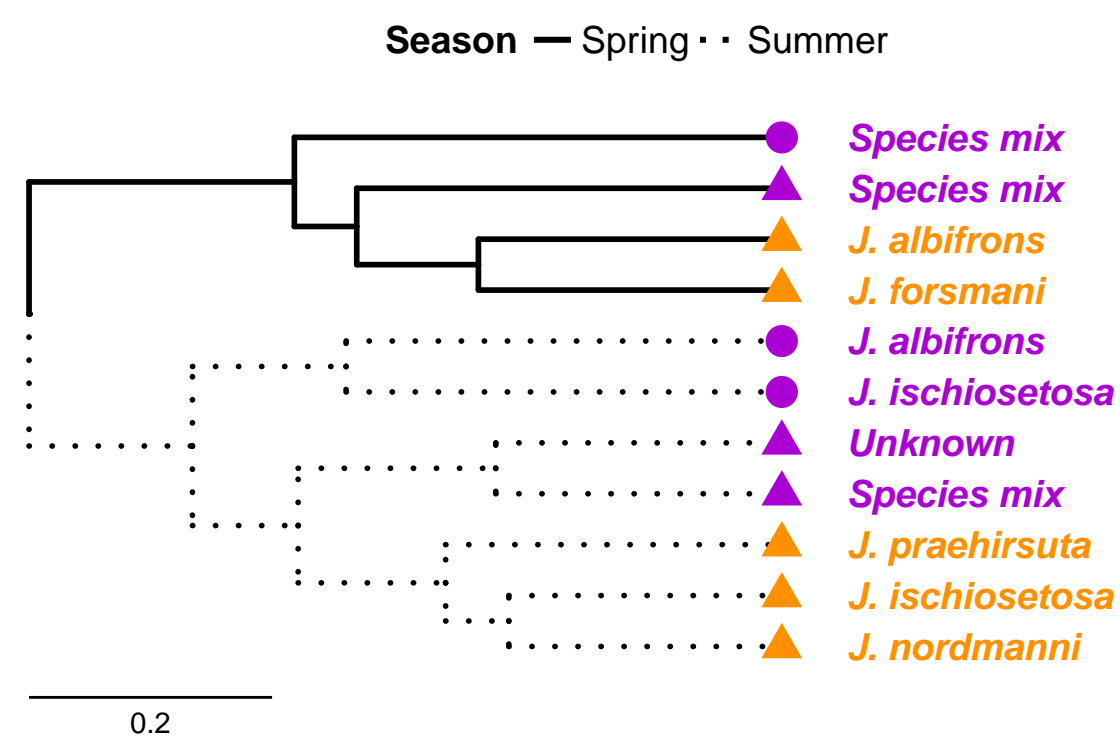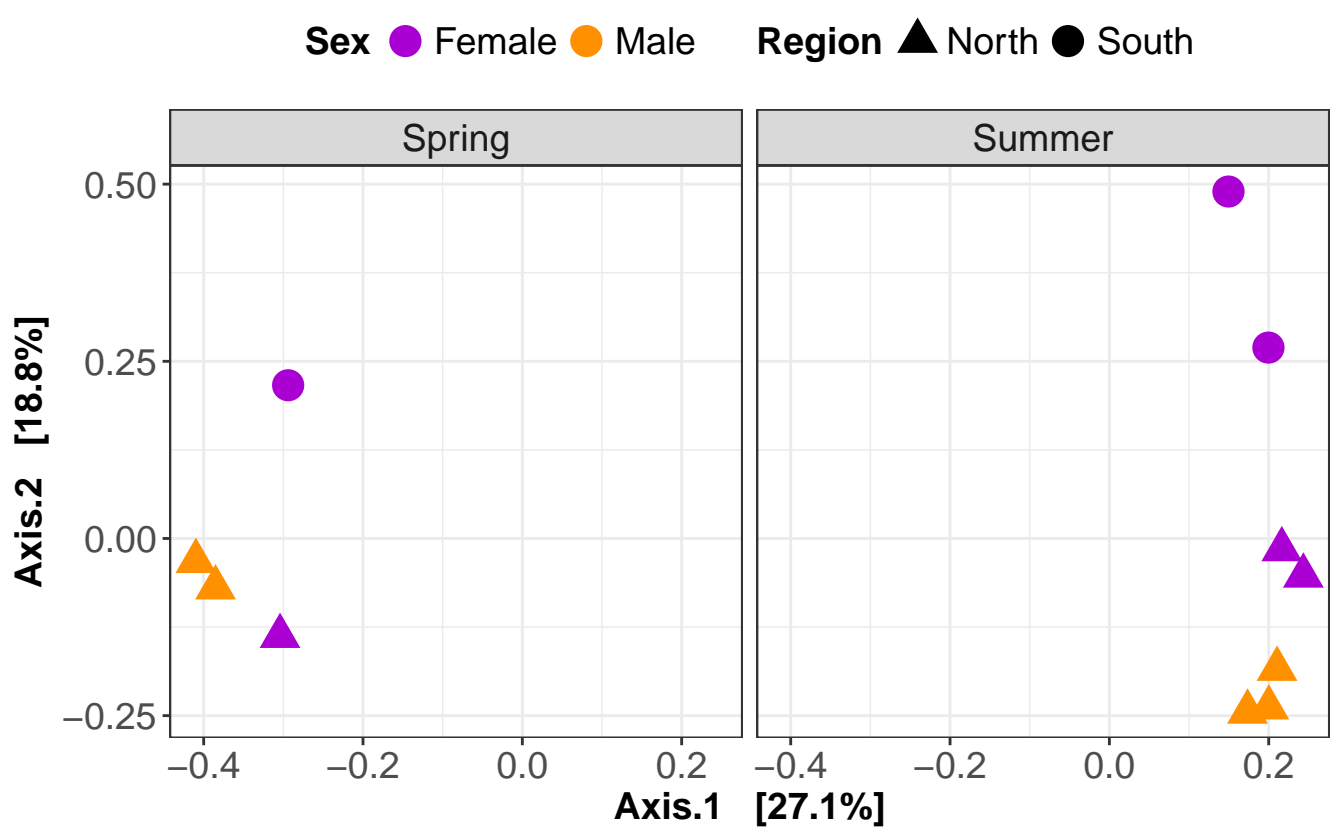

Bray-Curtis single-end

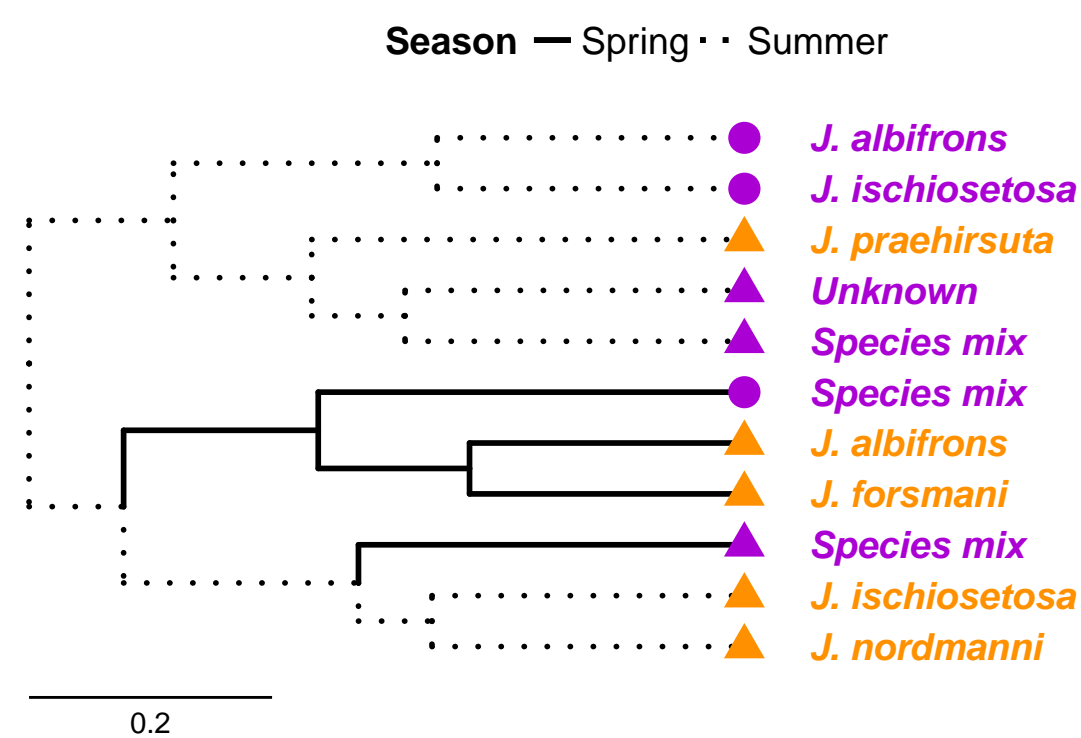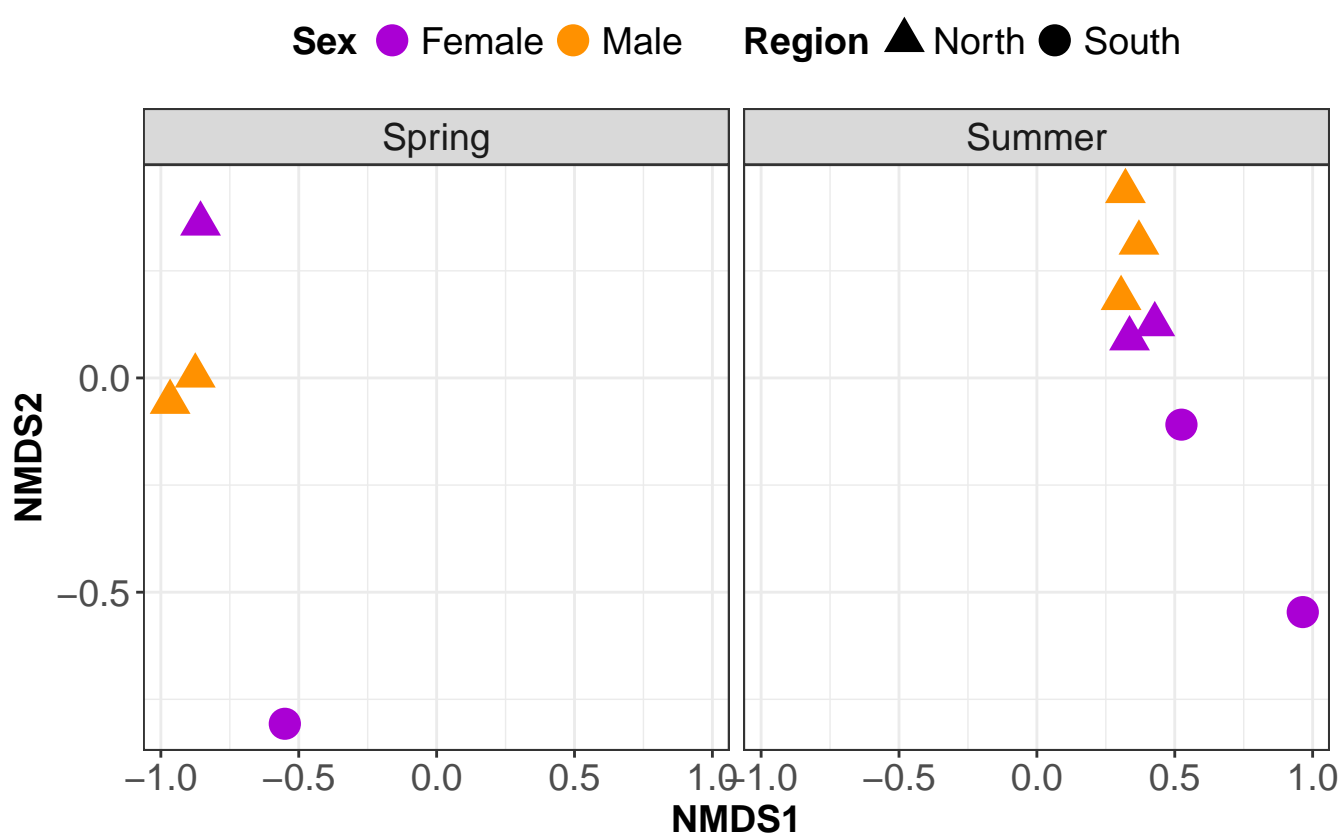

Bray-Curtis paired-end

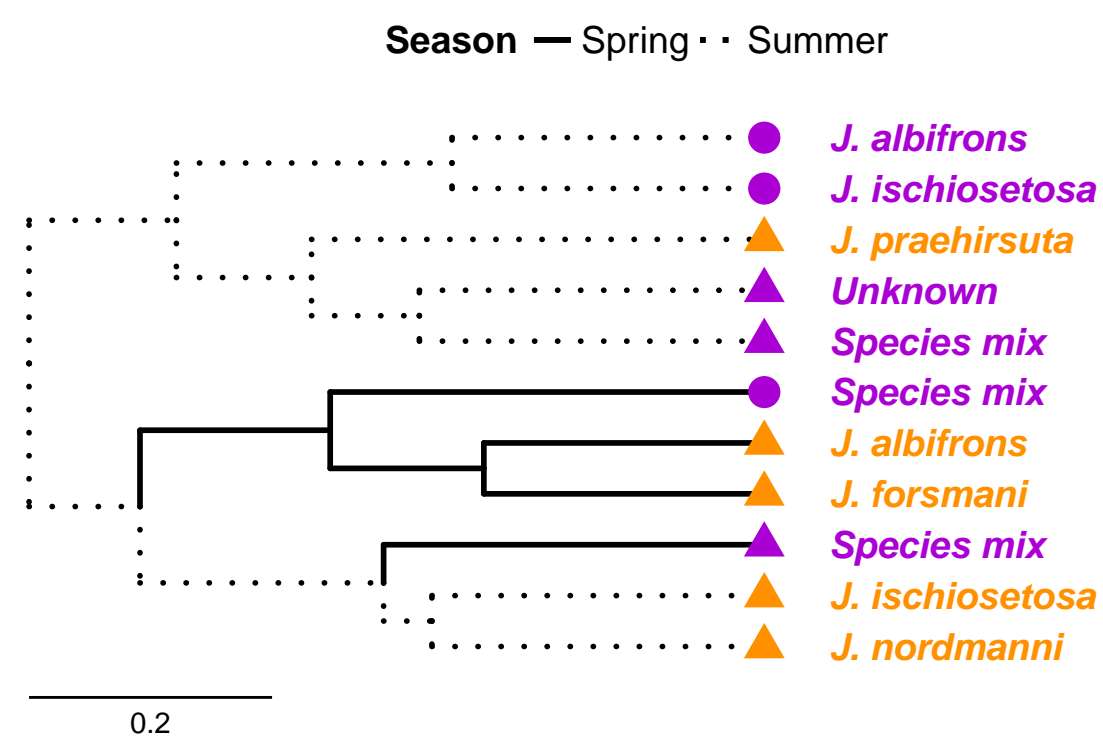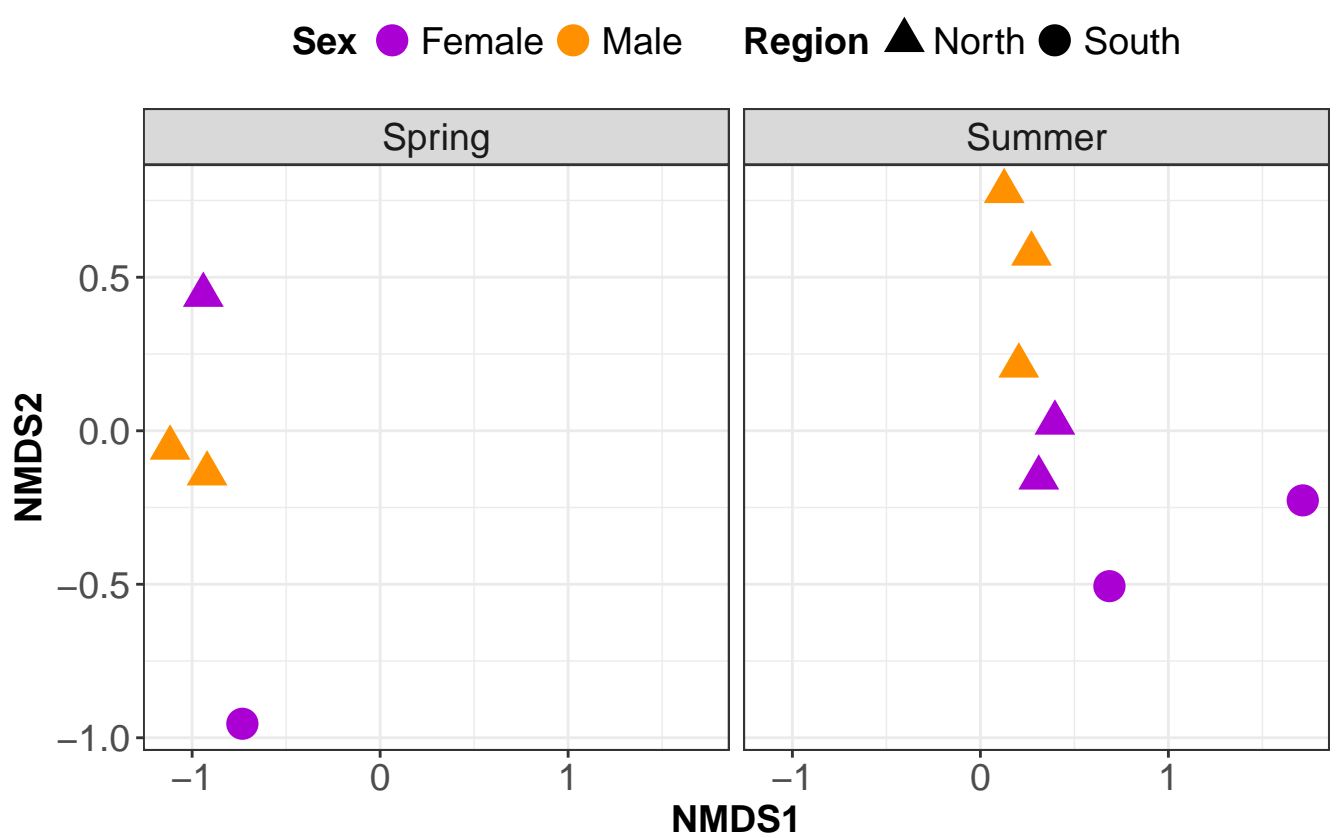

Supplement: S4 Fig — (PDF) [file pone.0202212.s004.pdf]

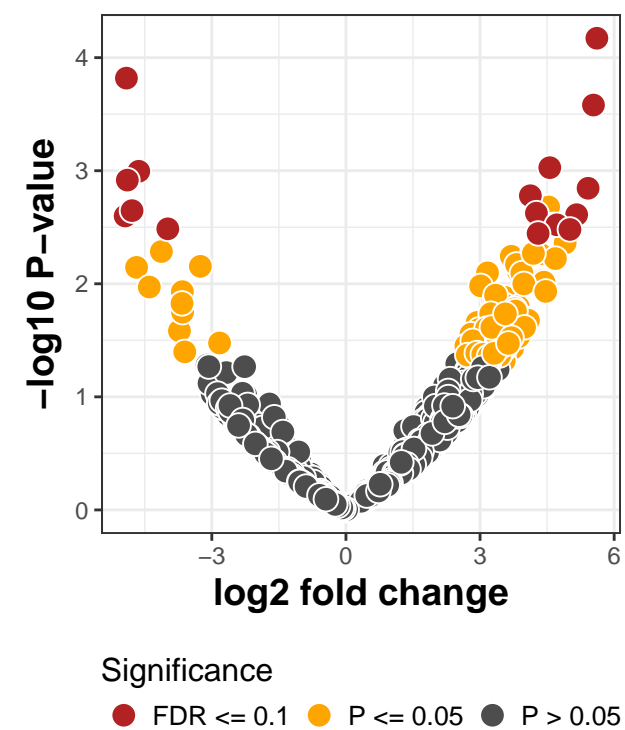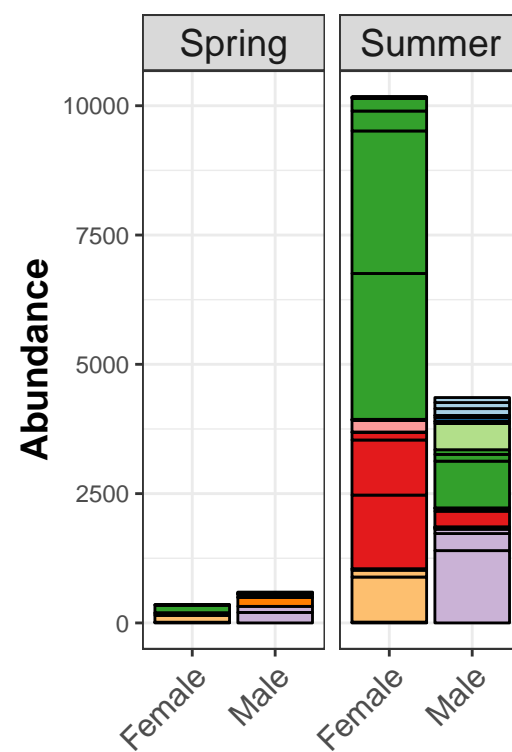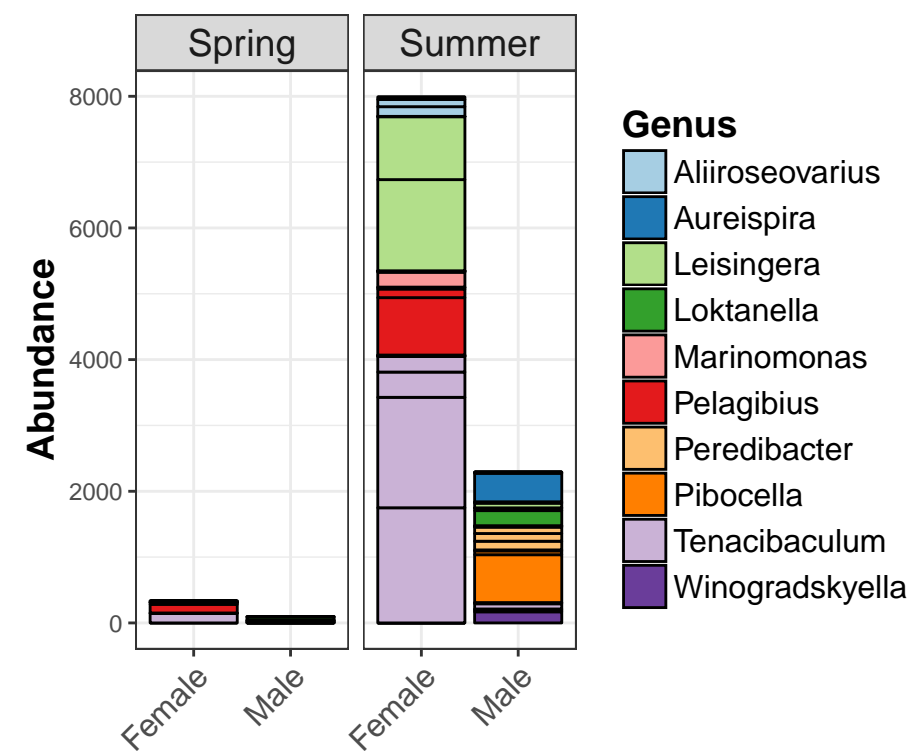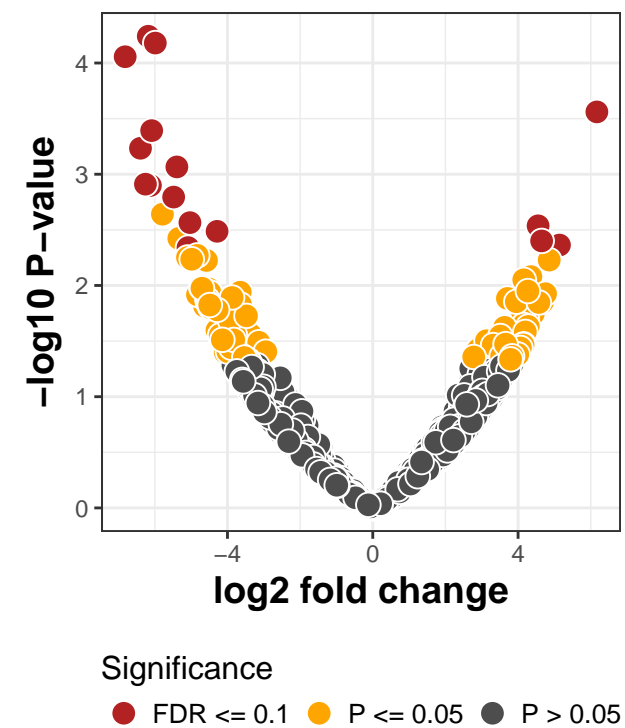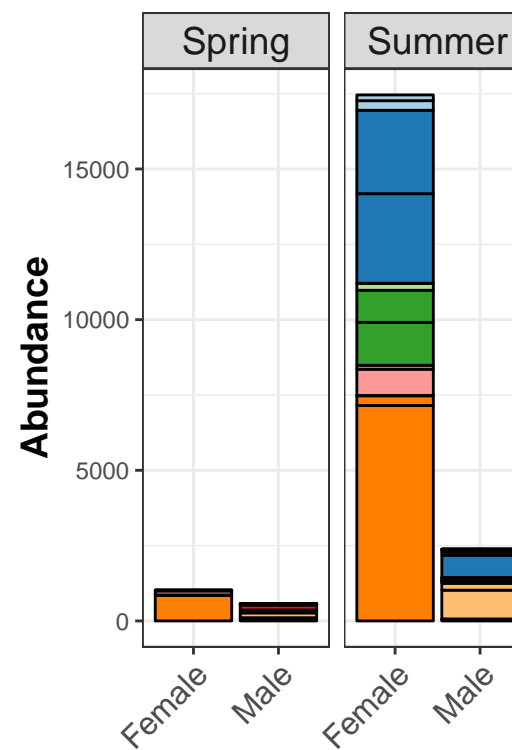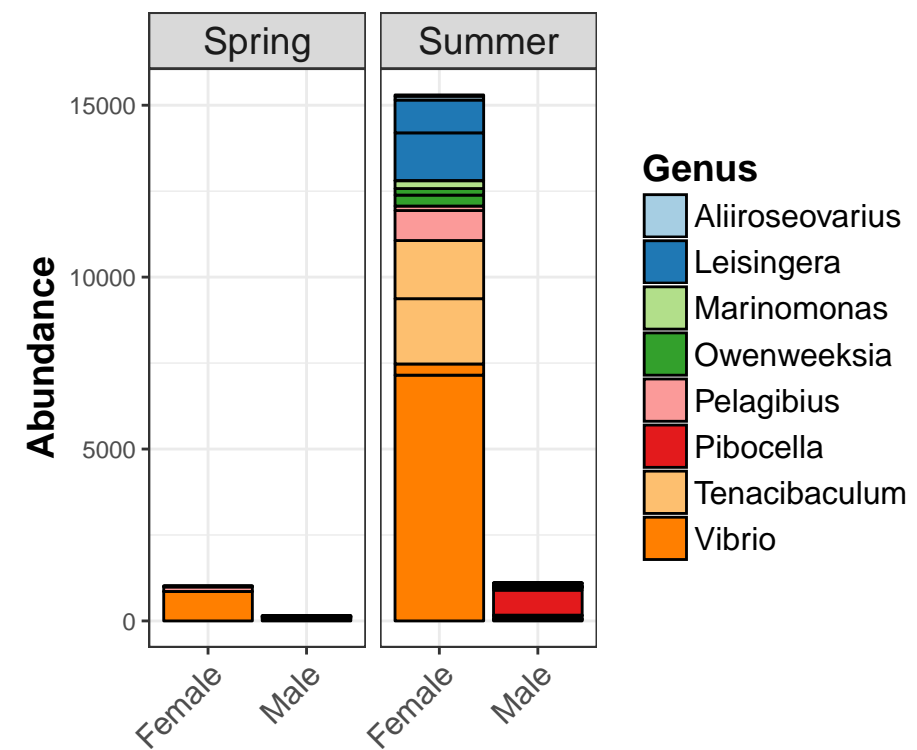

Supplement: S5 Fig — The top panels represent the full dataset of eleven samples; the bottom panels represent the eight samples from the northern region only. (PDF) [file pone.0202212.s005.pdf]

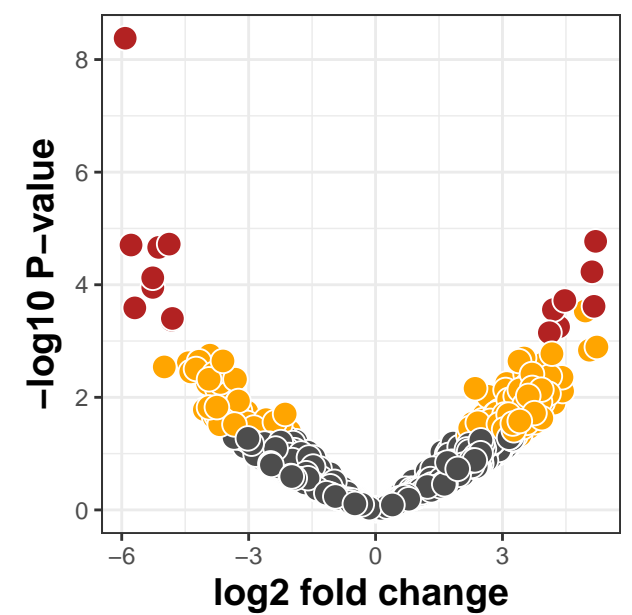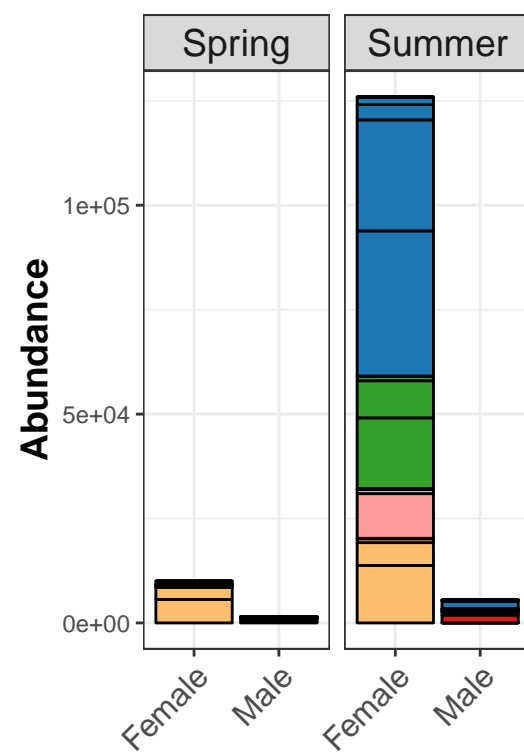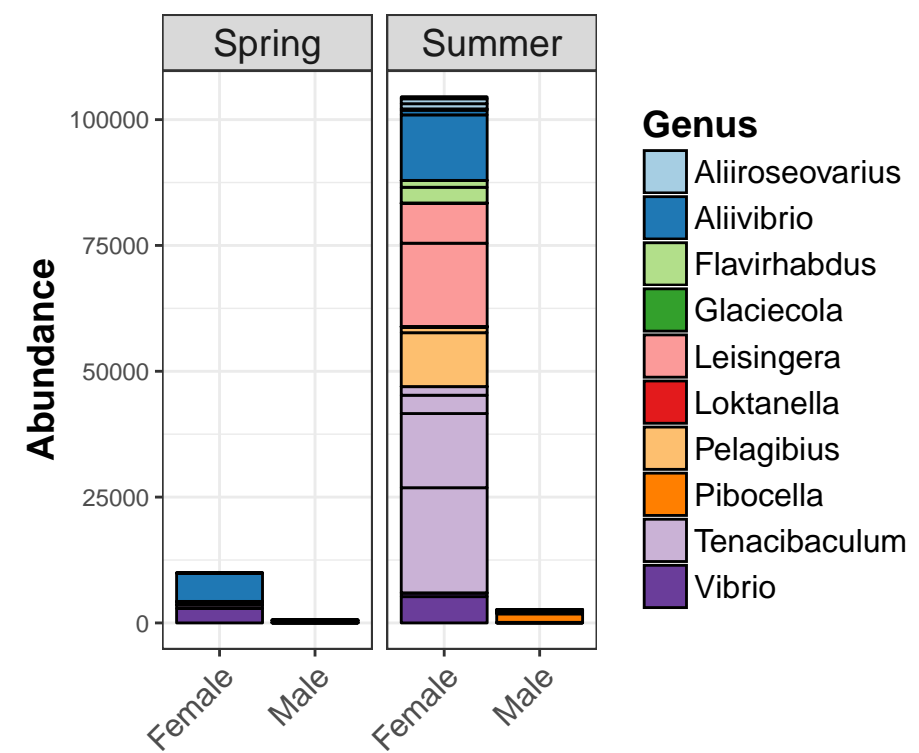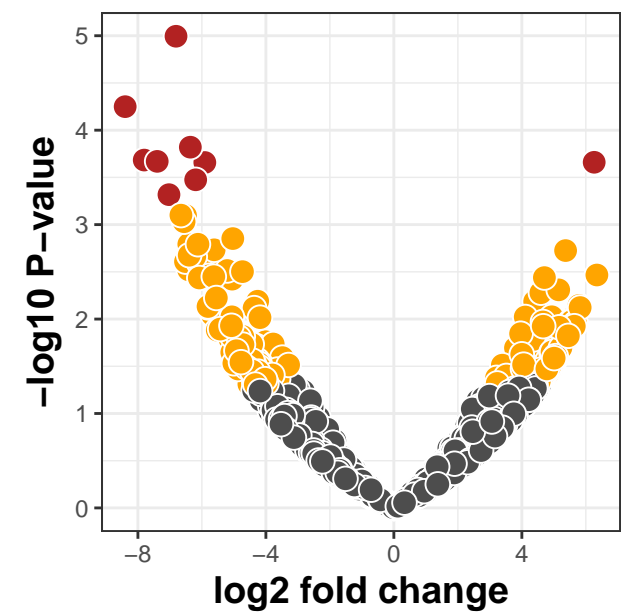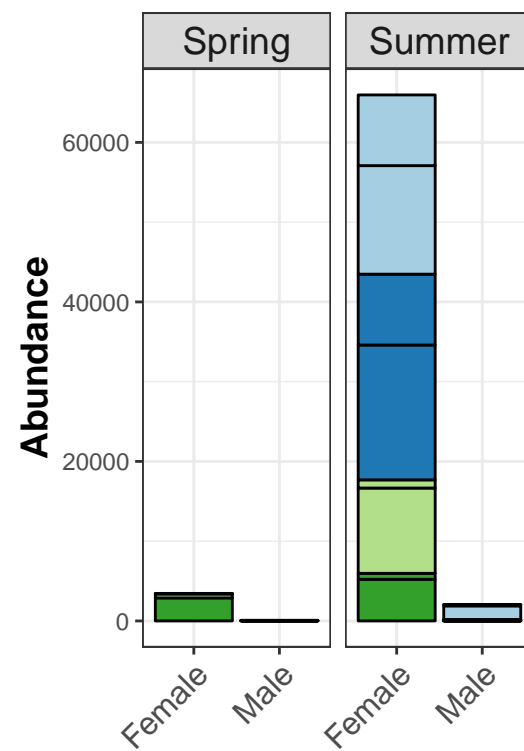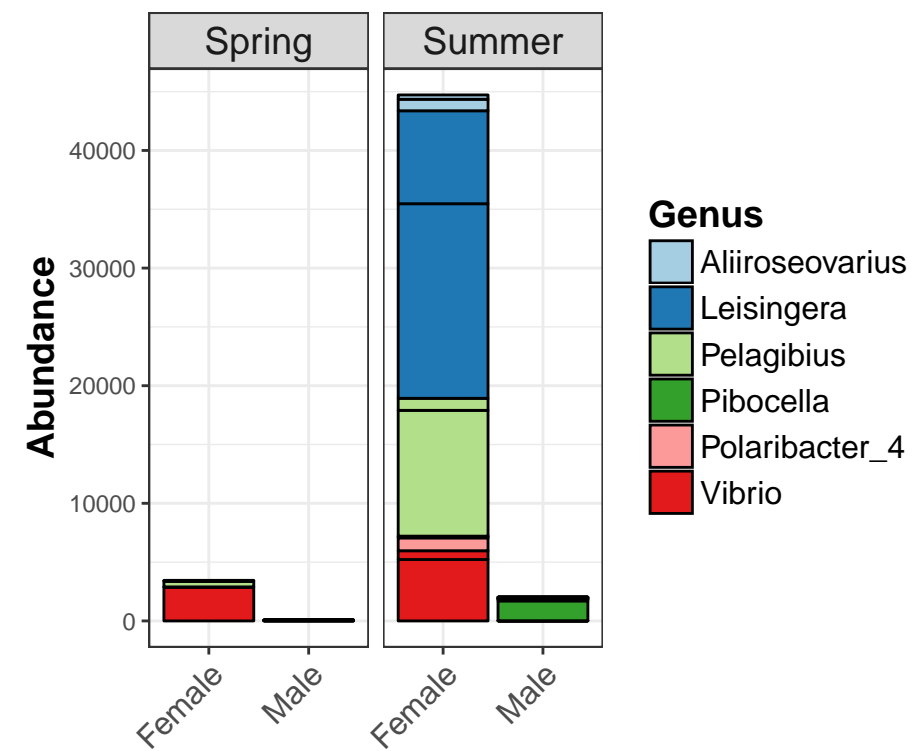

Supplement: S6 Fig — The top panels represent the full dataset of eleven samples; the bottom panels represent the eight samples from the northern region only. (PDF) [file pone.0202212.s006.pdf]
